# Causal relationship between gout and liver cancer: A Mendelian randomization and transcriptome analysis

**DOI:** 10.1097/MD.0000000000040299

**Published:** 2024-11-08

**Authors:** Jiaqi Xi, Xiaofang Cheng, Jun Liu

**Affiliations:** a Department of Endocrinology, People’s Liberation Army General Hospital of Southern Theatre Command, Guangzhou, China; b Department of Liver Injury and Repair, Guangxi Medical University, Nanning, China.

**Keywords:** gout, HCC, Mendelian randomization, PEMT

## Abstract

Gout is an inflammatory arthritis resulting from urate crystal deposition, now recognized as part of metabolic syndrome. Hyperuricemia, a hallmark of gout, is associated with various health complications, including liver cancer. Observational studies indicate a link between gout and increased cancer incidence. However, the causal relationship between gout and hepatocellular carcinoma remains uncertain. This study utilizes Mendelian randomization (MR) to explore this connection, minimizing confounding factors commonly present in observational studies. Genome-wide association study data for gout and liver cancer were sourced from the UK Biobank. We selected single nucleotide polymorphisms that are strongly associated with gout and liver cancer as instrumental variables for the analysis. We conducted 2-sample MR analysis using multiple MR methods (MR-Egger, weighted median, inverse variance weighting, and weighted mode) to evaluate causality. Co-localization and transcriptomic analyses were employed to identify target genes and assess their expression in hepatocellular carcinoma tissues. The 2-sample MR analysis indicated a significant causal relationship between gout and heightened liver cancer risk (*P*_IVW = .014). Co-localization analysis identified phosphatidylethanolamine N-methyltransferase (PEMT) as a crucial gene associated with gout (pH4 = 0.990). Transcriptomic data showed that PEMT expression was significantly higher in normal liver tissues compared to malignant samples (*P* < .001), and higher PEMT levels correlated with improved survival outcomes (*P* = .045). Immunohistochemical analysis revealed lower PEMT expression in hepatocellular carcinoma from patients with concurrent gout compared to those without (*P* < .05). The results indicate that gout increases the risk of hepatocellular carcinoma, with PEMT potentially playing a key role. Although this study focused on European populations, indicating a need for further research in diverse groups, the results emphasize the potential for liver cancer screening in newly diagnosed gout patients. Understanding the relationship between these conditions may inform future clinical practices and cancer prevention strategies.

## 1. Introduction

Gout is an inflammatory arthritis caused by the deposition of urate crystals in different joints.^[1]^ As the world economy progresses, the global incidence of gout has steadily increased to 7.9 per 1000 men and 2.5 per 1000 women.^[2]^ Sodium urate crystal deposition due to hyperuricemia is now widely recognized as the cause of gout.^[1]^ At the same time, increasing evidence suggests that gout is significantly associated with metabolic syndrome, with hyperuricemia playing an important role, which indicates that gout is also a metabolic disease.^[3]^ Liver cancer is the sixth most common cancer in the world, with a 5-year survival rate of about 18%.^[4]^ Viral hepatitis, alcoholic liver disease, and nonalcoholic fatty liver disease are important risk factors for liver cancer. Currently, nonalcoholic fatty liver disease is the leading cause of liver cancer in Western countries due to the increased prevalence rate of metabolic syndrome.^[5]^ Notably, multiple observational studies have shown that hyperuricemia or gout is associated with higher cancer incidence as well as mortality.^[6–10]^ In hepatocellular carcinoma, Guo et al^[11]^ found that hyperuricemia may affect the expression of PDZK1. PDZK1 affects proliferation, migration, and apoptosis of hepatocellular carcinoma through the STAT3/C-myc pathway. This suggests a potential association between gout and hepatocellular carcinoma. Gout is a chronic disease that can be prevented and controlled; therefore, understanding the relationship between gout and cancer risk is essential for reducing cancer mortality. However, the causal relationship between gout and liver cancer remains uncertain.

Mendelian randomization (MR) is a strategy that employs biologically meaningful genetic variation to examine causality,^[12]^ unlike previous observational studies, which were inconclusive due to key confounders like obesity, hypertension, diabetes mellitus, and heavy alcohol consumption. This method is commonly employed to investigate causal relationships between biological or medical variables,^[13]^ surpassing the constraints of conventional randomized controlled studies.^[14]^ It is a feasible approach for analyzing the causal link between gout and hepatocellular carcinoma. This work employed 2-sample MR to uncover the causative impact of gout on hepatocellular cancer. Furthermore, co-localization and transcriptomics analyses were utilized to confirm the findings of the MR analysis.

## 2. Materials and methods

### 2.1. Data sources and materials

The genome-wide association study (GWAS) data for gout (ukb-b-12765) comes from the UK Biobank (https://gwas.mrcieu.ac.uk/). Gout cases identified in the dataset are classified according to the International Classification of Diseases, 10th Edition code M10, which is defined as joint pain and swelling caused by the deposition of urate crystals. The code M10.99 indicates the presence of gout but does not specify which joint sites are affected. This dataset includes 1042 gout patients and 461,968 controls, with a total of 9851,867 single nucleotide polymorphisms (SNPs). The GWAS data for liver cancer (ieu-b-4953) also derives from the UK Biobank and was provided by Kimberley Burrows et al It includes 168 liver cancer patients and 372,016 controls, totaling 6304,034 SNPs. Detailed information about the data can be found in Table 1.

**Table 1:** Characteristics of GWAS summary data in Mendelian randomization studies.

Trait	GWAS ID	Year	Cases	Control	Sample size	SNPs	Population	Sex	Category
Diagnoses–secondary ICD-10: M10.99 Gout, unspecified (site unspecified)	ukb-b-12765	2018	1042	461,968	463,010	985,1867	European	Males and females	Binary
Liver cell carcinoma	ieu-b-4953	2021	168	372,016	372,184	630,4034	European	Males and females	Binary

GWAS = genome-wide association study, ICD-10 = International Classification of Diseases, 10th Edition, SNP = single nucleotide polymorphism.

Tumor tissues were collected from 10 patients with confirmed gout-free hepatocellular carcinoma and ten patients with confirmed gout combined with hepatocellular carcinoma from the First Affiliated Hospital of Guangxi Medical University, and these tissues were placed in liquid nitrogen immediately after surgical resection and then transferred to −80°C refrigerator for backup. All patients in the trial gave written informed consent, and the Ethics Committee of Guangxi Medical University reviewed and approved the study.

### 2.2. Variables of the screening tool

The selection of instrumental variables must meet the following criteria: choose SNPs that are strongly associated with the exposure factor, using (*P* < 5 × 10^−8^)as the inclusion criterion for significant association. Set the parameters (*R*² < 0.001) and linkage disequilibrium distance >10,000 kb, to remove linkage disequilibrium. Selecting SNPs with *F* > 10 to exclude weak instrumental variables. The calculation formula for the statistic *F* is as follows^[15]^:


$$\begin{array}{l} \begin{array}{l} F=\frac{N-K-1}{K}\times \frac{R^{2}}{1-R^{2}}\;\\ R^{2}=2\times \left(1-MAF\right)\times MAF\times \frac{\beta }{SD}\; \end{array} \end{array}$$

where N is the sample size of the exposure database, *K* is the number of SNPs, *R*² is the proportion of variation explained by the SNPs in the exposure database, MAF is the minor allele frequency, (*β*) is the effect size of the SNP on the exposure, and SD is the standard deviation.

### 2.3. Statistical analysis of MR

We used 2-sample MR analysis to investigate the causal link between gout and liver cancer. Gout is the exposure variable, cancer is the outcome variable, and SNP is the instrumental variable in the MR analysis. The 2-sample MR method assumes that the instrumental variable has a significant correlation with gout risk, influences liver cancer risk only through its impact on gout risk, and is not linked to any confounding variables. To examine the causal linkages, we employed 4 MR approaches, including MR-Egger, weighted median, inverse variance weighting (IVW), and weighted mode. *P* values below .05 were considered statistically significant. The IVW results were the primary basis, while other methods served as supplementary and validation tools.^[16]^ The *Q*-test (Cochran *Q*) was utilized to identify heterogeneity among instrumental variables, with a *P* value below .05 indicating its presence. The multiplicity of directions was evaluated based on intercepts from MR-Egger analyses,^[17]^ indicating that a *P* value under .05 suggested the presence of pleiotropy. The study used the R package “TwoSampleMR” and the online tool MRbase, available at (http://app.mrbase.org).^[18]^

### 2.4. Co-positioning analysis

Expression quantitative trait loci (eQTL) are genetic variants associated with gene expression phenotypes.^[19,20]^ We employed the co-localization analysis to pinpoint target genes associated with gout. The co-localization analysis integrates information from all eQTL SNPs, including cis and trans SNPs, by combining eQTL data from multiple tissues with GWAS data. When GWAS signaling and eQTL co-localization are identified, the GWAS locus may impact the expression phenotype of the target gene. We conducted co-localization analysis using GWAS data on gout “ukb-b-12765” from GTEx using the MR InstrumentsR package. We utilized the R package “coloc” for co-localization analysis.^[18]^ Due to the limited capacity of co-localization analysis, we confined the investigation to genes with pH4 ≥ 0.75.

### 2.5. Bioinformatics analysis

RNAseq data from the TCGA-LIHC project were downloaded from the TCGA database (https://portal.gdc.cancer.gov) in TPM format using R (v4.2.1). Clinical data were also extracted. The Welch *t* test was used to detect differences in phosphatidylethanolamine N-methyltransferase (PEMT) expression between hepatocellular carcinoma and normal liver tissues. Proportional risk hypothesis testing was conducted using the survival package, and survival regression was fitted. The results were visualized using the survival and ggplot2 packages. If an optimal grouping approach was chosen, optimal grouping cutoff screening was conducted appropriately using the surv_cutpoint function in the survival package.^[17]^ ROC analysis of the data was performed using the pROC tool, and the results were visualized using ggplot2, which analyzed gene enrichment between high and low-expression groups of PEMT using gene set enrichment analysis software (v2.10.1).^[21]^

### 2.6. Quantification of PEMT mRNA expression level using RT-qPCR

RNA was isolated from hepatocellular carcinoma tissues using the Trizol kit. The RNA was then reverse transcribed into cDNA using a reverse transcription kit. The expression level of PEMT mRNA was determined using RT-qPCR. The RT-qPCR reaction system consisted of 7.5 μL of 2 × Taq Green Master Mix, 1.5 μL each of forward and reverse primers, and 4.5 μL of cDNA. ⁃qPCR reaction conditions: predenaturation at 94°C for 3 minutes, denaturation at 94°C for 30 seconds, annealing at 55°C for 30 seconds, and extension at 72°C for 30 seconds, totaling 39 cycles. The relative expression of the target genes was analyzed using the 2−△△ method with β-actin as the internal reference gene. Comparisons between the 2 groups were made using the paired *t* test, which was statistically significant at *P* < .05, and the data were statistically analyzed using SPSS 21.0 software.

### 2.7. The expression level of PEMT in hepatocellular carcinoma was determined via immunohistochemical analysis

Hepatocellular carcinoma tissue sections were deparaffinized using xylene and gradient alcohol, followed by citrate buffer (pH = 6.0) treatment and microwave antigen repair. The sections were then incubated with PEMT antibody at 4°C overnight, followed by the addition of a rabbit secondary antibody. Color development was achieved using 3,3’-diaminobenzidine for 5 seconds, followed by hematoxylin restaining for 30 seconds. The sections were rinsed, dehydrated, air-dried, and sealed before being observed using an immunohistochemistry scanning instrument to preserve the corresponding results. The scoring system for staining intensity is as follows: 0 points for no staining, 1 point for mild staining, 2 points for moderate staining, and 3 points for severe staining. The scoring system for the staining area is as follows: 0 points for no staining, 1 point for <30% of cells stained, 2 points for 30% to 60% of cells stained, and 3 points for more than 60% of cells stained. Positive PEMT staining is indicated by brown staining in the cytoplasm. PMET expression was evaluated by combining the scores for staining intensity and staining area. Comparisons between the 2 groups were made using the paired *t* test, which was statistically significant at *P* < .05, and the data were statistically analyzed using SPSS 21.0 software.

## 3. Result

### 3.1. Results of MR analysis

SNPs were utilized as instrumental variables in a 2-sample MR analysis, with gout as the exposure and liver cancer as the outcome. The results indicated a significant causal relationship between gout and a high risk of hepatocellular carcinoma (*P*MR-Egger = .533, *P*_weighted-median = .033, *P*_IVW = .014, and *P*_weighted-mode = .221; Table 2). The heterogeneity assessment found little evidence of heterogeneity in the association (Cochran *Q*_MR-Egger = 0.907 and *P* = .341; *Q*_IVW = 1.212 and *P* = .546; Table 3). Horizontal pleiotropy analysis indicated minimal evidence for the association (*P* = .679; Table 4).

**Table 2:** Two-sample MR results of gout as the exposure and liver cancer as the outcome.

Exposure	Outcome	Method	SNP	*β*	SE	*P*
Gout	Liver cell carcinoma	MR-Egger	3	0.2396	0.266	.5332
		Weight median	3	0.09578	0.04495	.0331
		IWW	3	0.09422	0.03823	.01371
		Weight mode	3	0.09871	0.05624	.2213

IWW = Instrumental Variable Weighting.

**Table 3:** Heterogeneity test.

Heterogeneity test	Method	*Q*	*Q*_df	*Q*_*P*val
	MR-Egger	0.9066	1	.341
	IWW	1.212	2	.5456

IWW = Instrumental Variable Weighting.

**Table 4:** Test for directional horizontal pleiotropy.

Test for directional horizontal pleiotropy	Egger_intercept	SE	*P*
	−0.00011	2e-04	.679

### 3.2. Identifying target genes for gout by co-localization analysis

**Figure 1.**
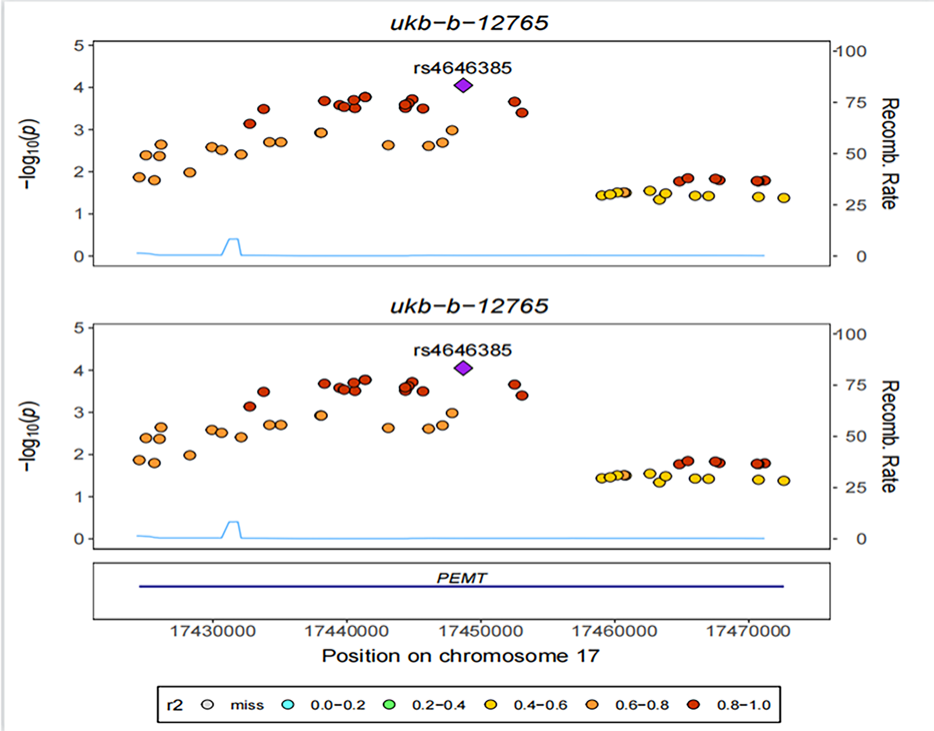
eQTL data and gout co-localization analysis of correlation region Manhattan map. (A) rs4646385 (purple dot) represents the SNP with the highest correlation of eQTL data with gout. eQTL = expression quantitative trait locus, SNP = single nucleotide polymorphism.

eQTLs are genetic variations linked to gene expression traits. When co-localization analysis was performed using GTEx eQTL data and GWAS data for gout (ukb-b-12765), PEMT was shown to be a gene substantially linked with gout (pH4 = 0.990; Fig. 1).

### 3.3. Upregulation of PEMT is linked to the prognosis of hepatocellular cancer

**Figure 2.**
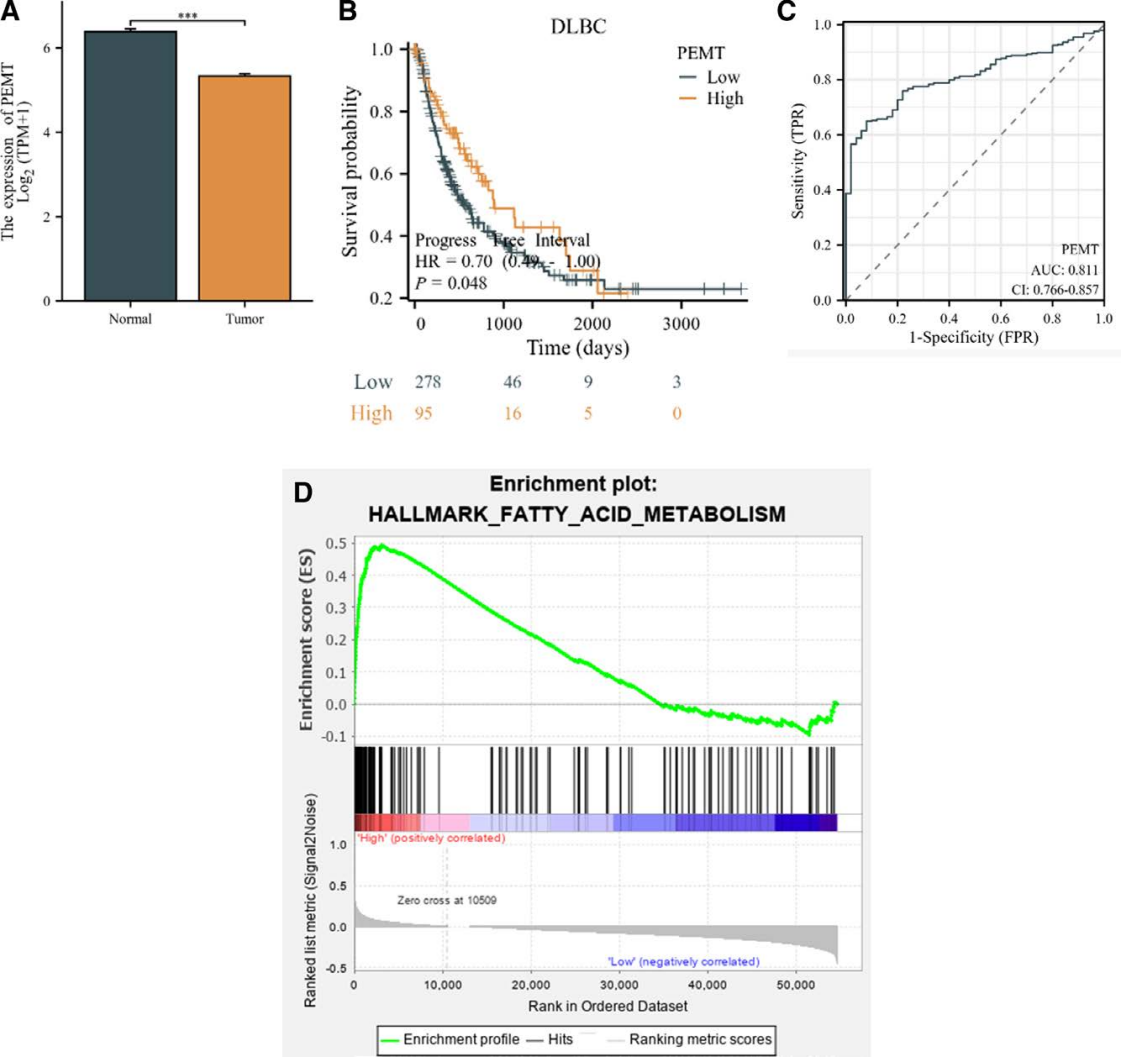
Relative expression of PEMT in HCC, impact on survival and diagnostic value and potential pathways. (A) Expression of PEMT in HCC in the TCGA dataset. (B, C) Impact of PMET on survival and diagnostic value of HCC in the TCGA dataset. (D) GSEA analysis of potential pathways of action of PEMT in HCC. GSEA = gene set enrichment analysis, HCC = hepatocellular carcinoma, PEMT = phosphatidylethanolamine N-methyltransferase.

PEMT expression was higher in normal liver tissues compared to malignant tissue samples (*P* < .001; Fig. 2A). The high PEMT expression group had a superior prognosis for progression-free survival compared to the low PEMT expression group (*P* = .045; Fig. 2B). Furthermore, the area under curve is frequently utilized to assess diagnostic tests. The area under curve value for PEMT was 0.811 (Fig. 2C), indicating its diagnostic importance. The potential involvement of PEMT in HCC was investigated through gene set enrichment analysis, revealing a significant correlation between PEMT and fatty acid metabolic pathways (NES = 1.756, *P* = .01; Fig. 2D).

### 3.4. Low levels of PEMT expression in hepatocellular carcinoma tissues from people diagnosed with gout

**Figure 3.**
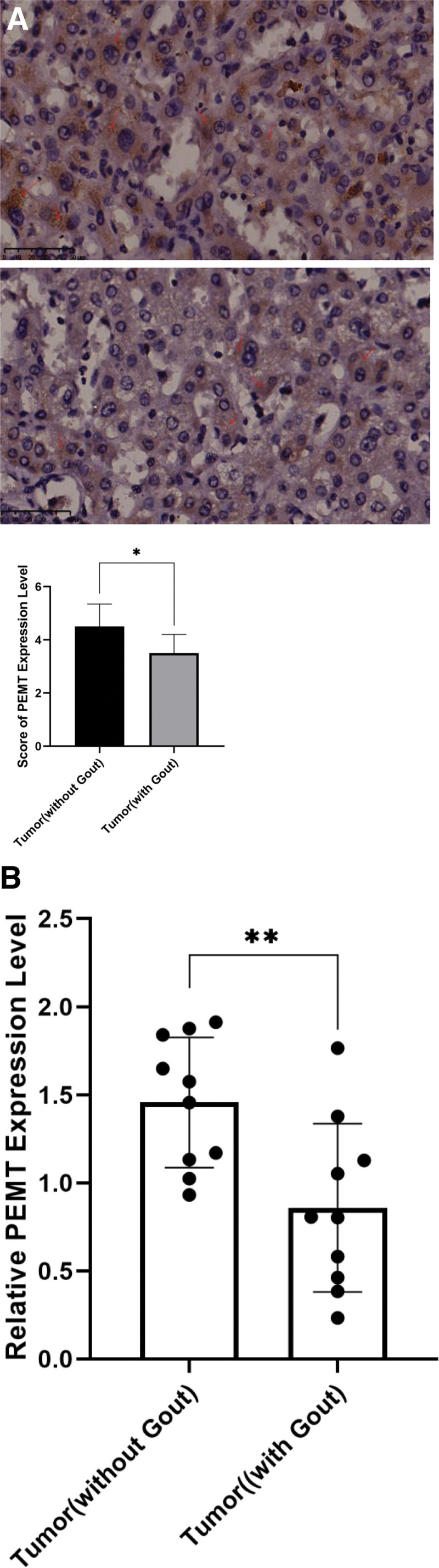
PEMT expression in hepatocellular carcinoma tissues. (A) Immunohistochemical analysis of PEMT expression in hepatocellular carcinoma tissues and score statistics. (B) RT-qPCR assessment of PEMT mRNA expression in hepatocellular carcinoma tissues. *P-value of < 0.05; **P-value of < 0.01. PEMT = phosphatidylethanolamine N-methyltransferase, RT-qPCR = reverse transcription-quantitative polymerase chain reaction.

Immunohistochemical tests and RT-qPCR revealed a notable decrease in PEMT expression in hepatocellular carcinoma tissues of patients with combined gout compared to those with regular hepatocellular carcinoma (*P* < .05; Fig. 3A and *P* < .01; Fig. 3B).

## 4. Discussion

We examined the causal link between gout and hepatocellular cancer in a substantial sample of GWAS public data using a 2-sample MR approach. The findings indicated that a genetic predisposition to gout is a risk factor for liver cancer. Furthermore, we pinpointed PEMT as a crucial gene influencing gout characteristics through co-localization analysis. Transcriptome research revealed that PEMT, which was downregulated in hepatocellular carcinoma, may contribute to the suppression of this cancer and is positively associated with progression-free survival. PEMT may serve as a crucial link connecting gout and cancer.

Gout may play a role in the onset of liver cancer through specific pathways. Research is currently investigating this connection to gain a better understanding. Gout is a condition resulting from disruptions in the metabolism of purine-like compounds, with hyperuricemia being the primary biochemical foundation and the most significant risk factor for developing gout.^[1]^ Studies indicate that patients with gout may have a higher risk of developing colon, breast, biliary tract, and liver cancers.^[22]^ Elevated levels of uric acid in the blood may be an early sign of cancer development and are associated with a higher risk of cancer-related death.^[6]^ Managing uric acid levels through medication could potentially reduce cancer mortality rates.^[23]^ Nevertheless, the precise connection between serum uric acid levels and cancer mortality remains uncertain. Reports indicate that individuals with gout between the ages of 41 and 55 have a greater likelihood of developing cancer compared to those without gout.^[8]^ Ministrini et al^[24]^ documented a case of gout stones in the liver, which was subsequently followed by hepatocellular cancer at the same location. Reports on the correlation between gout and the risk of liver cancer are few, making it a promising area for further investigation.

Many gout patients exhibit various cancer risk factors, such as heavy alcohol intake, smoking, obesity, and metabolic syndrome, which complicate the evaluation of the direct link between gout and cancer risk. This issue was effectively circumvented in this study using the MR procedure. Nevertheless, this study has limitations as the GWAS data for gout and liver cancer are derived from European populations, and their generalizability to other regions requires further investigation. Second, although confounding factors were avoided, the variability in the diagnoses and symptoms of gout and hepatocellular carcinoma made the collected data unrepresentative of all European populations affected by gout, and gender- and age-specific analyses were not undertaken. Magnetic resonance imaging results could only partially clarify the linear connection between gout and hepatocellular carcinoma. Nonlinear studies were not feasible, and further epidemiological methods are required to validate the association between gout and hepatocellular carcinoma. Finally, we did not experimentally verify the cancer-inhibitory effect of PEMT.

Individuals newly diagnosed with gout may have an elevated risk of developing liver cancer, making it crucial for them to undergo screening for liver cancer. This study demonstrates a direct impact of gout on liver cancer, suggesting a possible biological connection between the 2 conditions.

## Acknowledgments

The authors are grateful to the UK Biobank, TCGA database, and all participants in our study.

## Author contributions

**Writing—review & editing:** Jiaqi Xi, Jun Liu.

**Data curation:** Xiaofang Cheng.

## References

[1] Dalbeth N Merriman TR Stamp LK. Gout. Lancet. 2016;388:2039–52. 27112094 10.1016/S0140-6736(16)00346-9

[2] Danve A Neogi T. Rising global burden of gout: time to act. Arthritis Rheumatol. 2020;72:1786–8. 33150696 10.1002/art.41453 PMC7644950

[3] Thottam GE Krasnokutsky S Pillinger MH. Gout and metabolic syndrome: a tangled web. Curr Rheumatol Rep. 2017;19:60. 28844079 10.1007/s11926-017-0688-y

[4] Kotsari M Dimopoulou V Koskinas J Armakolas A. Immune system and hepatocellular carcinoma (HCC): new insights into HCC progression. Int J Mol Sci. 2023;24:11471. 37511228 10.3390/ijms241411471 PMC10380581

[5] Chavez-Tapia NC Murua-Beltran GS Ordonez-Vazquez AL Nuno-Lambarri N Vidal-Cevallos P Uribe M. Understanding the role of metabolic syndrome as a risk factor for hepatocellular carcinoma. J Hepatocell Carcinoma. 2022;9:583–93. 35818404 10.2147/JHC.S283840 PMC9270896

[6] Xie Y Xu P Liu K. Hyperuricemia and gout are associated with cancer incidence and mortality: a meta-analysis based on cohort studies. J Cell Physiol. 2019;234:14364–76. 30693505 10.1002/jcp.28138 PMC13482836

[7] Boffetta P Nordenvall C Nyren O Ye W. A prospective study of gout and cancer. Eur J Cancer Prev. 2009;18:127–32. 19337060 10.1097/CEJ.0b013e328313631a

[8] Lee JS Myung J Lee HA. Risk of cancer in middle-aged patients with gout: a nationwide population-based study in Korea. J Rheumatol. 2021;48:1465–71. 33191287 10.3899/jrheum.200497

[9] Chen CJ Yen JH Chang SJ. Gout patients have an increased risk of developing most cancers, especially urological cancers. Scand J Rheumatol. 2014;43:385–90. 24825466 10.3109/03009742.2013.878387

[10] Gremke N Griewing S Kostev K Wagner U Kalder M. Association between gout and subsequent breast cancer: a retrospective cohort study including 67,598 primary care patients in Germany. Breast Cancer Res Treat. 2023;199:545–52. 37071268 10.1007/s10549-023-06944-w PMC10175324

[11] Guo L Jiang W Quan L Teng X Zhao J Qiu H. Mechanism of PDZK1 in hepatocellular carcinoma complicated with hyperuricemia. J Oncol. 2022;2022:1403454. 36420358 10.1155/2022/1403454 PMC9678461

[12] Emdin CA Khera AV Kathiresan S. Mendelian randomization. JAMA. 2017;318:1925–6. 29164242 10.1001/jama.2017.17219

[13] Yu H Song X. The relationship between Alzheimer disease and thyroiditis: a two-sample Mendelian randomization study. Medicine (Baltim). 2023;102:e35712. 10.1097/MD.0000000000035712 PMC10627647 37933065

[14] Sekula P Del GMF Pattaro C Kottgen A. Mendelian randomization as an approach to assess causality using observational data. J Am Soc Nephrol. 2016;27:3253–65. 27486138 10.1681/ASN.2016010098 PMC5084898

[15] Jiang R Mou S Luo F Zhang Z. Causal relationship between chronic obstructive pulmonary disease and BMD at different sites: a bidirectional Mendelian randomization study. Medicine (Baltim). 2023;102:e35495. 10.1097/MD.0000000000035495 PMC10578729 37832103

[16] Hartwig FP Davies NM Hemani G Davey SG. Two-sample Mendelian randomization: avoiding the downsides of a powerful, widely applicable but potentially fallible technique. Int J Epidemiol. 2016;45:1717–26. 28338968 10.1093/ije/dyx028 PMC5722032

[17] Burgess S Thompson SG. Erratum to: Interpreting findings from Mendelian randomization using the MR-Egger method. Eur J Epidemiol. 2017;32:391–2. 28527048 10.1007/s10654-017-0255-x PMC5506233

[18] Dong Z Xu M Sun X Wang X. Mendelian randomization and transcriptomic analysis reveal an inverse causal relationship between Alzheimer’s disease and cancer. J Transl Med. 2023;21:527. 37542274 10.1186/s12967-023-04357-3 PMC10403895

[19] Liu Y Li B Ma Y Huang Y Ouyang F Liu Q. Mendelian Randomization Integrating GWAS, eQTL, and mQTL data identified genes pleiotropically associated with atrial fibrillation. Front Cardiovasc Med. 2021;8:745757. 34977172 10.3389/fcvm.2021.745757 PMC8719596

[20] Zuber V Grinberg NF Gill D. Combining evidence from Mendelian randomization and colocalization: review and comparison of approaches. Am J Hum Genet. 2022;109:767–82. 35452592 10.1016/j.ajhg.2022.04.001 PMC7612737

[21] Ai H. GSEA-SDBE: a gene selection method for breast cancer classification based on GSEA and analyzing differences in performance metrics. PLoS One. 2022;17:e0263171. 35472078 10.1371/journal.pone.0263171 PMC9041804

[22] Kuo CF Luo SF See LC Chou IJ Fang YF Yu KH. Increased risk of cancer among gout patients: a nationwide population study. Joint Bone Spine. 2012;79:375–8. 22088929 10.1016/j.jbspin.2011.09.011

[23] Yang HC Nguyen P Islam M. Gout drugs use and risk of cancer: a case-control study. Joint Bone Spine. 2018;85:747–53. 29427783 10.1016/j.jbspin.2018.01.008

[24] Ministrini S Baronio G Zorzi F. Unusual presentation of gouty tophus in the liver with subsequent appearance in the same site of HCC: a correlate diagnosis? Case report. World J Surg Oncol. 2019;17:10. 30621724 10.1186/s12957-018-1546-8 PMC6325729

